# Efficacy and safety results from the first pivotal phase 3 randomized controlled trial of mdma-assisted psychotherapy for treatment of severe chronic PTSD

**DOI:** 10.1192/j.eurpsy.2021.391

**Published:** 2021-08-13

**Authors:** B. Van Der Kolk

**Affiliations:** School Of Medicine, Boston University, West Stockbridge, United States of America

**Keywords:** neuroplasticity, MDMA, psychotherapy, ptsd

## Abstract

**Introduction:**

Posttraumatic stress disorder is a prevalent mental health condition with substantial impact on daily functioning that lacks sufficient treatment options. Previous research has led to the designation of 3,4-methylenedioxymethamphetamine (MDMA) as a Breakthrough Therapy for treatment of post-traumatic stress disorder (PTSD) when administered as an adjunct to psychotherapy.

**Objectives:**

Here we report the findings of the first randomized, double-blind, Phase 3 trial assessing the efficacy and safety of 3 sessions with a flexible dose of MDMA or placebo administered under direct observation to participants with severe PTSD (n = 100) as an adjunct to inner-directed psychotherapy.

**Methods:**

Change in PTSD symptoms (CAPS-5) and functional impairment (SDS) were assessed by a central, blinded Independent Rater Pool at baseline and following each treatment session. Adverse events (AEs), concomitant medications, suicidal ideation and behavior were tracked throughout the study. Vital signs were measured during experimental sessions. The primary endpoint was 18 weeks post-randomization.

**Results:**

Change in CAPS-5 and SDS, placebo-subtracted Cohen’s d effect size, and a responder analysis will be presented. There were three serious AEs of suicidal ideation or behavior reported. MDMA was well tolerated, with some treatment emergent AEs occurring at greater frequency for the MDMA group during and after experimental sessions.

**Conclusions:**

If MDMA-assisted psychotherapy significantly attenuates PTSD symptomatology and associated functional impairment, these results will form the basis for marketing authorization applications worldwide, including among participants with dissociative subtype of PTSD, depression, history of alcohol and substance use disorders, and adverse childhood experiences.

**Disclosure:**

No significant relationships.

